# Reconstruction of the Hepatic Microenvironment and Pathological Changes Underlying Type II Diabetes through Single-Cell RNA Sequencing

**DOI:** 10.7150/ijbs.99176

**Published:** 2024-10-14

**Authors:** Chia-Yen Dai, Ying-Ming Tsai, Chao-Yuan Chang, Hung-Pei Tsai, Kuan-Li Wu, Yu-Yuan Wu, Ling-Yu Wu, Shu-Fang Jian, Pei-Hsun Tsai, Chai-Tung Ong, Chien-Hui Sun, Ya-Ling Hsu

**Affiliations:** 1 School of Medicine, College of Medicine, Kaohsiung Medical University, Kaohsiung 807, Taiwan.; 2 Division of Hepato/Billiary/Pancreatic, Kaohsiung Medical University Hospital, Kaohsiung Medical University, Kaohsiung 807, Taiwan.; 3 Division of Pulmonary and Critical Care Medicine, Kaohsiung Medical University Hospital, Kaohsiung Medical University, Kaohsiung 807, Taiwan.; 4 Department of Anatomy, Kaohsiung Medical University, Kaohsiung 807, Taiwan.; 5 Division of Neurosurgery, Department of Surgery, Kaohsiung Medical University Hospital, Kaohsiung 807, Taiwan.; 6 Graduate Institute of Medicine, College of Medicine, Kaohsiung Medical University, Kaohsiung 807, Taiwan.; 7 Drug Development and Value Creation Research Center, Kaohsiung Medical University, Kaohsiung 807, Taiwan.; 8 National Pingtung University of Science and Technology, Department of Biological Science and Technology, Pingtung, 912, Taiwan.

**Keywords:** T2DM, MAFLD, Tgfb1i1, Txnip, Capillarization

## Abstract

The global prevalence of type 2 diabetes mellitus (T2DM) continues to rise. Therefore, it has become a major concern health issue worldwide. T2DM leads to various complications, including metabolic-associated fatty liver disease (MAFLD). However, comprehensive studies on MAFLD as a diabetic complication at different stages are still lacking. Using advanced single-cell RNA-seq technology, we explored changes of livers in two T2DM murine models. Our findings revealed that increase activation of hepatic stellate cells (HSCs) exacerbated the development of MAFLD to steatohepatitis by upregulating transforming growth factor β1 induced transcript 1 (*Tgfb1i1*). Upregulated thioredoxin-interacting protein (*Txnip*) contributed to hepatocyte damage by impairing reactive oxygen species clearance. Additionally, the capillarization of liver sinusoidal endothelial cells correlated with *Fabp4* overexpression in endothelial cells. A novel subset of Kupffer cells (KCs) that expressed *Cd36* exhibited an activated phenotype, potentially participating in inflammation in the liver of diabetic mice. Furthermore, ligand-receptor pair analysis indicated that activated HSCs interacted with hepatocytes or KCs through* Thbs2* and *Lamb2* in late-stage diseases. The reduction in cell-cell interactions within hepatocytes in diabetic mice, reflects that the mechanisms regulating liver homeostasis is disrupted. This research underscores the importance of dynamics in diabetic MAFLD, and provides new insights for targeted therapies.

## Introduction

The overall occurrence of metabolic-associated fatty liver disease (MAFLD) in the global adult population is estimated to be around 25%. This escalating prevalence of MAFLD is contributing to various liver diseases, such as non-alcoholic steatohepatitis (NASH), fibrosis, hepatic cirrhosis, and finally hepatocellular carcinoma (HCC) 1. Diabetes represents an important risk factor for the development by driving the progression of liver injury from simple steatosis to NASH and the subsequent liver fibrosis. Epidemiological studies reveal that up to 70% of individuals with type 2 diabetes mellitus (T2DM) concurrently experience MAFLD 2, 3. Death rates from vascular events have declined in both diabetic and non-diabetic populations, whereas death rates from liver disease have increased exclusively among individuals with T2DM 4. Both T2DM and MAFLD involve subtle yet significant changes that can cause disability and various metabolic issues 5. Each one independently heightens the risks of mortality and morbidity, together intensifying the burden on worldwide financial and healthcare systems 6. Therefore, it is crucial to ascertain how diabetes-related pathogenic factors, including hyperglycemia, insulin resistance, and glucose and lipid metabolic dysregulation, elevate the risk of severe forms of MAFLD.

The liver is composed of four major types of cells, including hepatocytes, liver sinusoidal endothelial cells (LSECs), Kupffer cells (KCs), and hepatic stellate cells (HSCs) that collectively contribute to its homeostasis, functions and disease development 7. Hepatocytes serve as the primary functional unit of the liver, crucial for energy metabolism, detoxification, and protein synthesis due to their abundance of mitochondria, rough endoplasmic reticulum (ER), Golgi apparatus, and free ribosomes. With remarkable regenerative capabilities, hepatocytes play a significant role in proliferation following liver injury 8. Lipotoxicity resulting from excessive lipid accumulation in hepatocytes due to metabolic dysregulation promotes oxidative and ER stress, metabolic inflammation, hepatocyte ballooning, apoptosis, and cell death 9. Hepatocyte loss, inflammation, and metabolic changes induce the transformation of quiescent HSCs into activated myofibroblast-like cells (activated HSCs), which exhibit proliferation, migration, and contractile properties 9, 10. Dysfunction of LSECs is also considered as a primary and early event in the progression of MAFLD, and it plays a key role in hampering hepatic lipid uptake and metabolism, disrupting the transport of macromolecules and metabolites, influencing angiogenesis, fostering intrahepatic inflammation, causing hepatocellular damage, and ultimately leading to impaired hepatic blood flow with increased intrahepatic resistance 11, 12. An elevated number of KCs/macrophages have been noted in association with the presence of sizable lipid droplets, showing a positive correlation with the severity of MAFLD. In addition, an increase in the accumulation of endolysosomal lipids in KCs is observed during steatosis to NASH progression, suggesting a key role of lipids in KCs activation and their impact on MAFLD progression 13. Understanding the intricate interplay among the diverse cell types in fatty liver underscores the complexity of hepatic responses to metabolic disturbances.

The hepatic microenvironment contains permeable connective tissue that facilitates biological exchange between portal blood flow from the gastrointestinal tract and hepatocytes. Within this niche, bi- or tri-communications occurs among various neighboring cell types, hepatocyte, immune cells, and LSECs through soluble mediators. These interactions may ultimately lead to the initiation and progression of fibrosis through intricate processes involving nearby cells, predominantly hepatocytes, LSECs, KCs, and HSCs 1, 14. Advancements in single cell RNA Sequencing (scRNA-seq) technology offer an unprecedented opportunity to dissect the liver's cellular landscape at a single-cell resolution. By elucidating these interactions, we aim to export a comprehensive understanding of the dynamics in liver microenvironment during the disease progress in fatty liver. This nuanced insight may pave the way for targeted therapeutic strategies tailored to specific cellular responses within the liver, ultimately advancing our ability to mitigate the impact of fatty liver disease.

## Materials and Methods

### Experimental animals

Two murine models were utilized to study diabetes in this research. The first model involved C57BL/KsJ-db/m (control, male, n=6) and C57BL/KsJ-db/db (diabetic, male, n=6) mice, with liver samples collected at 22 weeks old (mid-stage) and 33 weeks old (late-stage) for scRNA-seq analysis. The second model comprised a regimen of a high-fat diet (HFD) followed by streptozotocin (STZ) administration, using C57BL/6 mice. Male mice (n=6) aged eight weeks were fed with an HFD (60% fat) for 12 weeks, subsequently receiving an STZ intraperitoneal injection at a dosage of 100 mg/kg. These mice were euthanized at 28 weeks old, and their livers were harvested and preserved by formalin-fixed paraffin-embedded (FFPE) for histological examination. Glucose monitoring was conducted via tail vein sampling. All subjects were procured from the National Animal Center and the study protocols were sanctioned by the Institutional Animal Care and Use Committee (IACUC) at Kaohsiung Medical University (IACUC no. 110131).

### scRNA-seq analysis

FFPE liver blocks collected from db/m and db/db, control, and HFD/STZ mice were cut into thickness as 50 μm curls. And then they were dissociated using with the Miltenyi Biotech FFPE Tissue Dissociation Kit (Cat no.130-118-052) following demonstrated protocol CG000233 ((10X Genomics, Pleasanton, CA, USA). For the Chromium workflows, cells were loaded onto the Chromium X instrument, adhering to the protocols detailed in the Chromium Fixed RNA Profiling for Multiplexed Samples (CG000527). cDNA libraries were sequenced on an Illumina NextSeq2000 (San Diego, CA, USA) using paired-end dual-indexing. The sequencing runs for all libraries were demultiplexed with bcl2fastq (Illumina) and processed with Cell Ranger v7.1.0 (10x Genomics), employing the Cell Ranger count and Cell Ranger pipeline on each gene expression microbead well with the mm10-2020A reference to produce gene-barcode matrices and other outputs. Gel bead-in emulsion wells were subsequently aggregated with the Cell Ranger pipeline for detailed analysis.

### Bioinformatics

Signature genes had to be expressed in more than 50% of the cells in at least one of the cell groups, with gene expression changes exceeding a 2-fold increase (log2 FC > 1). The pathway enrichment analysis was identified through Kyoto Encyclopedia of Genes and Genomes (KEGG) and Gene Set Enrichment Analysis (GSEA). Additionally, to explore the genes potentially contributed to cell transition or differentiation, trajectory analysis was carried out using Monocle 2 (version 2.22). A comprehensive database of known cytokine/chemokine receptor and ligand pairs was curated for analyzing cell-cell interactions. The database integrates data from several sources: CellTalkDB (http://tcm.zju.edu.cn/celltalkdb/), CellPhoneDB (www.cellphonedb.org/) and CellChat (github.com/sqjin/CellChat). These resources, combined with additional insights from previously published literature, provided a robust foundation for detailed examination of cytokine and chemokine-mediated communication between cells.

### Cell culture

LX-2 cells, a human HSC cell line, were maintained in Dulbecco's Modified Eagle Medium (DMEM), supplemented with 2% fetal bovine serum (FBS) and glutamine (2 mM) at 37 ℃ in a humidified atmosphere containing 5% CO_2_. AML-12 cells, a mouse hepatocyte cell line, were cultured in DMEM/F-12 medium enriched with 10% FBS, insulin (10 μg/ml), transferrin (5.5 μg/ml), selenium (5 ng/ml) and dexamethasone (40 ng/ml). Human Hepatic Sinusoidal Endothelial Cells (HHSECs, Catalog No. 5000, Sciencell, Carlsbad, CA,) were cultured in endothelial cell growth medium (ECGM, Catalog No. 1001, Sciencell), supplemented with 5% of FBS (Catalog No. 0025), 5 ml of Endothelial Cell Growth Supplement (ECGS, Catalog No. 1052) and 5 ml of penicillin/streptomycin solution (Catalog No. 0503). All cell lines were passaged upon reaching approximately 80% of confluence to maintain optimal growth and viability. HHSECs were not passaged more than six times to preserve their phenotypic characteristics. To simulate diabetic stress conditions, the cell lines LX-2, AML-12, and HHSECs were exposed to different treatment regimens: normal glucose (NG, 5.5 mM), high glucose (HG, 25 mM), palmitic acid (PA, 50 µM), and advanced glycation end products (AGEs, 300 µg/ml, cat no: 121800, Sigma-Aldrich, St. Louis, MO, USA). Additionally, co-treatment scenarios were employed, combining PA with HG and PA with AGEs for 48 h. Subsequently, the cells were harvested for analysis in downstream specialized assays.

### Western blotting/ELISA

Western blotting was performed to measure protein levels. Cells were first homogenized in the radio-immunoprecipitation assay lysis buffer supplemented with protease inhibitors. After centrifugation, protein concentrations were quantified using bicinchoninic acid assay. Proteins were resolved by sodium dodecyl sulfate-polyacrylamide gel electrophoresis and electrotransferred to polyvinylidene difluoride membranes. Membranes were blocked with 5% non-fat milk in Tris-buffered saline with Tween 20 (0.1%) for 1 hour at room temperature, then probed with primary antibodies overnight at 4 ℃. This was followed by incubation with horseradish peroxidase-linked secondary antibodies for 1 hour at room temperature. Detection was achieved using enhanced chemiluminescence. For quantification, band intensities were analyzed with ImageJ software, normalizing to glyceraldehyde 3-phosphate dehydrogenase. The details of the primary antibodies used in this study were specified in Table S1. The levels of S100A10 and laminin β2 protein in the serum or supernantant of LX2 cells were assessed using mouse S100A10 (Novus biologicals, Littleton, CO, USA) and laminin β2 (cat# ab288588, Abcam) ELISA kits.

### Txnip knockdown by siRNA transfection

The Txnip ON-TARGET plus SMARTpool siRNA and the non-targeting siCONTROL siRNA were sourced from Dharmacon (Lafayette, CO, USA). Cell transfections were carried out using DharmaFECT 3 transfection reagent (Dharmacon), following the protocol provided by the manufacturer. In brief, both the siRNA and the transfection reagent were diluted in serum-free Opti-MEM and then mixed. After a 20-minute incubation at room temperature, the mixture was applied to the cells at a final siRNA concentration of 20 nM. After 6 hours of incubation, FBS was added to reach a final concentration of 10%, and the cells were further incubated for 24 hours before undergoing subsequent treatments. The knockdown efficacy was validated by Western blot.

### Reactive oxygen species (ROS) detection

AML-12 cells were seeded in 96-well plates and treated under normal glucose (5.5 mM), HG (25 mM), PA (50 µM), or AGE (300 µg/ml) for 24 or 48 hours experimental conditions. After treatment, ROS levels was assessed using ROS-Glo H_2_O_2_ assay (Promega, Madison, WI, USA) following the standard protocol. Luminescence intensity, indicative of ROS levels, was measured using a luminescence microplate reader, which provides a quantitative measure of ROS accumulation in cells in response to various stimuli.

### Immunohistochemistry (IHC) and Oil Red O staining

IHC was conducted to detect protein localization in paraffin-embedded tissue sections. Sections of livers were deparaffinized and rehydrated, followed by antigen retrieval in citrate. The sections were blocked with 5% bovine serum albumin and incubated with primary antibodies overnight at 4 ℃. Following washes in phosphate-buffered saline, sections were incubated with horseradish peroxidase-conjugated secondary antibodies for 1 hour at room temperature. Visualization was achieved using either 3,3'-diaminobenzidine (DAB) or Emerald chromogen, and sections were counterstained with hematoxylin. Slides were then dehydrated, cleared, and mounted for microscopic analysis. The details of the primary antibodies used in this study were specified in Table S1.

Formalin-fixed liver sections underwent hematoxylin-eosin and Masson's trichrome staining for pathological examination. The evaluation of MAFLD was conducted using the MAFLD Activity Score (MAS). This scoring system assigns individual scores for steatosis (ranging from 0 to 3), hepatocellular ballooning (ranging from 0 to 2), and lobular inflammation (ranging from 0 to 3). To evaluate lipid accumulation in the liver, we performed Oil Red O staining on 5 μm thick cryosections of liver tissue. Sections were fixed in 10% formalin for 10 minutes, and then stained with Oil Red O for 15 minutes to highlight lipid droplets. Subsequently, sections were counterstained with hematoxylin to delineate nuclei and mounted for microscopic examination.

### Quantification and statistical analysis

Each *in vitro* experiment was performed independently and replicated at least three times to confirm reproducibility. For statistical comparisons between two groups, unpaired, two-tailed Student's t-tests were used. For analyses involving more than two groups, one-way Analysis of Variance (ANOVA) was employed, followed by Tukey's *post hoc* test to adjust for multiple comparison. All statistical analyses were conducted using Prism 9 software (GraphPad Software version 9.0, Prism). A significance level of *p*-value less than 0.05 was set for all tests.

## Results

### Two diabetes-related fatty liver models

**Figure 1:**
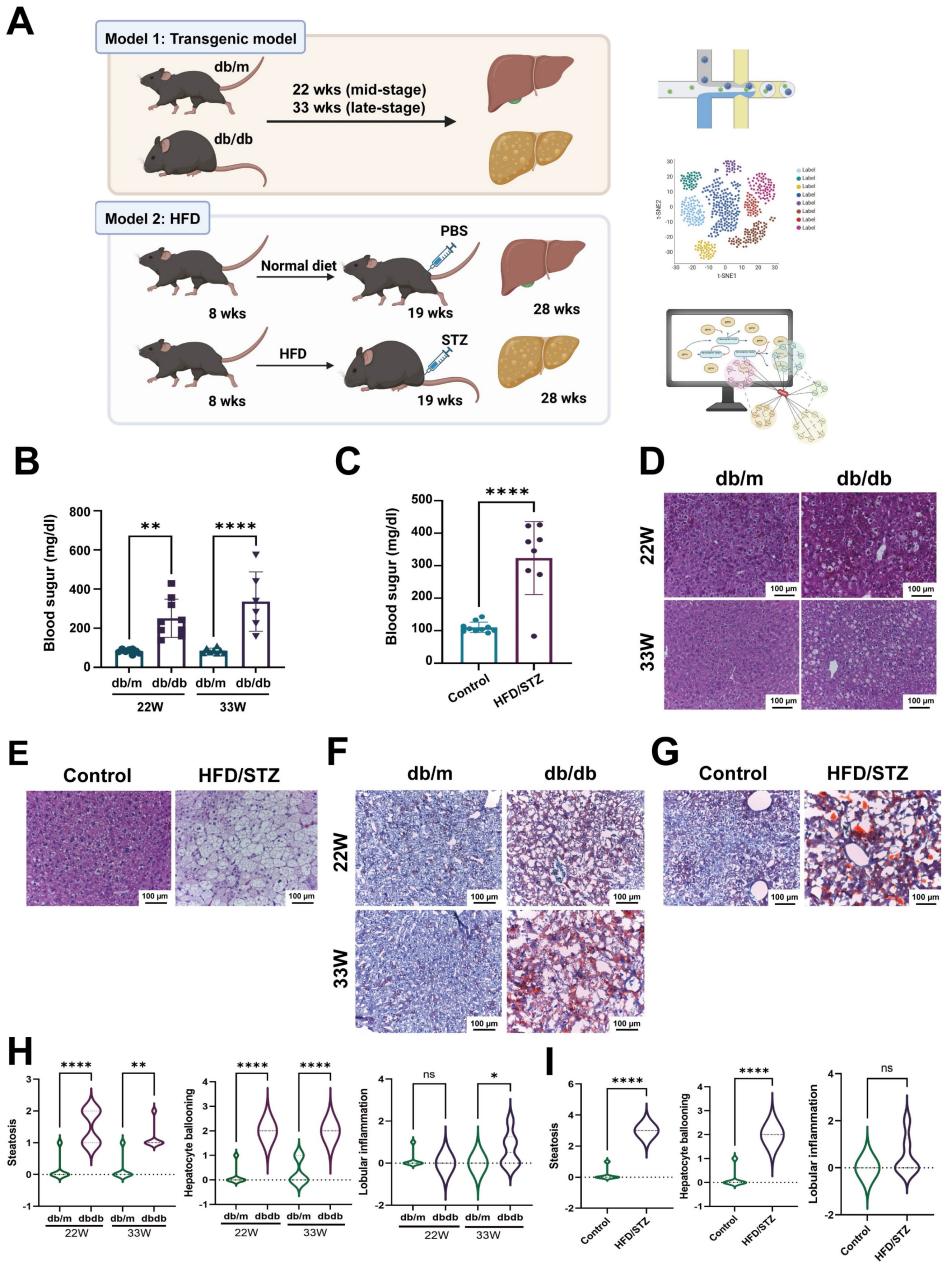
S**chematic representation of scRNA-seq pipeline using two diabetic models.** (A) The flowchart of scRNA-seq analysis of livers harvested from two diabetic mice models. The levels of blood sugar of db/m and db/db mice (B) or HFD/STZ mice (C). The H&E staining of livers of db/m and db/db mice (D) or HFD/STZ mice (E). The oil red staining of livers of db/m and db/db mice (F) or HFD/STZ mice (G). The development of hepatic steatosis, hepatocyte ballooning and lobular inflammation in db/m and db/db mice (H) and HFD/STZ(I) models **, p < 0.01, ****, p < 0.001. Abbreviations: HFD, high-fat diet; STZ, streptozotocin; PBS, phosphate-buffered saline.

To explore the mechanisms of diabetic fatty liver, we employed two diabetic animal models: db/db mice and HFD/STZ model. To investigate the changes at mid- and late-stages, we collected liver samples at 22 weeks (mid-stage diabetes) and 33 weeks (late-stage diabetes) for scRNA-seq to analyze the transcriptome profiles (Figure 1A). Blood sugar levels showed that both db/db mice and HFD/STZ model met the diabetes criteria (> 250 mg/dl) 15 (Figure 1B,C). HE staining results also revealed significant lipid droplet accumulation and vacuolization in the liver (Figure 1D,E), with more severe vacuolization observed at 33 weeks old compared with 22 weeks old. Oil Red O staining indicated substantial lipid accumulation in the liver (Figure 1F,G). These results suggested that both db/db mice and HFD/STZ model were optimal for studying diabetic fatty liver. As shown in Fig 1H, the db/db mice developed hepatocyte steatosis, ballooning, and scattered inflammatory cell infiltration. The MAFLD activity score was also significantly higher in the HFD/STZ model, compared with the liver of control mice (Figure 1I).

### The liver cell profile of mice with T2DM cross two models

**Figure 2:**
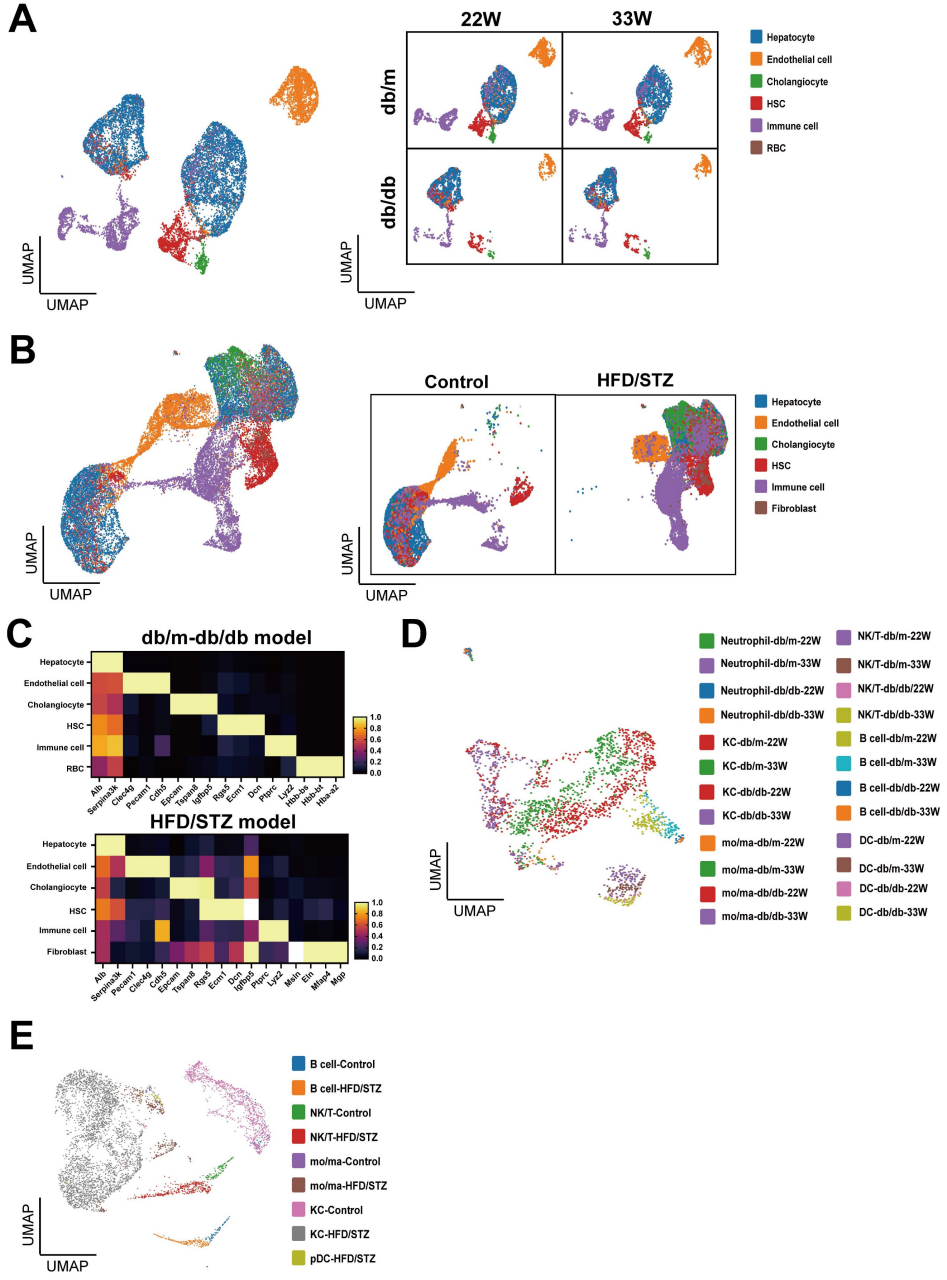
** Clustering and annotation of single-cell transcriptomes of liver in mice with T2DM.** (A) Uniform Manifold Approximation and Projection (UMAP) visualization of livers in db/m and db/db mice at 22W and 33W of age. (B) UMAP visualization of mice liver cells treated with or without HFD/STZ. (C) The cell markers used to cluster main cell subpopulations. The cell clusters of immune cells of db/m and db/db mice (D) or HFD/STZ mice (E). Abbreviations: HSC, hepatic stellate cell; RBC, red blood cell, KC, Kupffer cell; DC, dendritic cell; mo/ma, monocyte/macrophage.

We used the Uniform Manifold Approximation and Projection (UMAP) to analyze and visualize liver cell clusters in mice. In the db/db mice, five principal clusters were identified in db/db mouse model: Hepatocytes, Endothelial cells (ECs), Cholangiocytes, Hepatic stellate cells (HSCs), and Immune cells (Figure 2A). Meanwhile, six main cell clusters were defined in the HFD/STZ model: Hepatocytes, ECs, Cholangiocytes, HSCs, Immune cells, and Fibroblasts (Figure 2B), which were characterized using specific cell markers (Figure 2C). We reclustered immune cells and identified 6 major populations, including neutrophil, Kupffer cell (KC), NK/T cell, B cell, monocyte/macrophage (mo/ma), and dendritic cell (DC) (Figure 2D,E).

### Evaluated HSCs activation by *Tgfb1i1* and *Lamb2* upregulation in the late-stage fatty liver of diabetic mice

**Figure 3:**
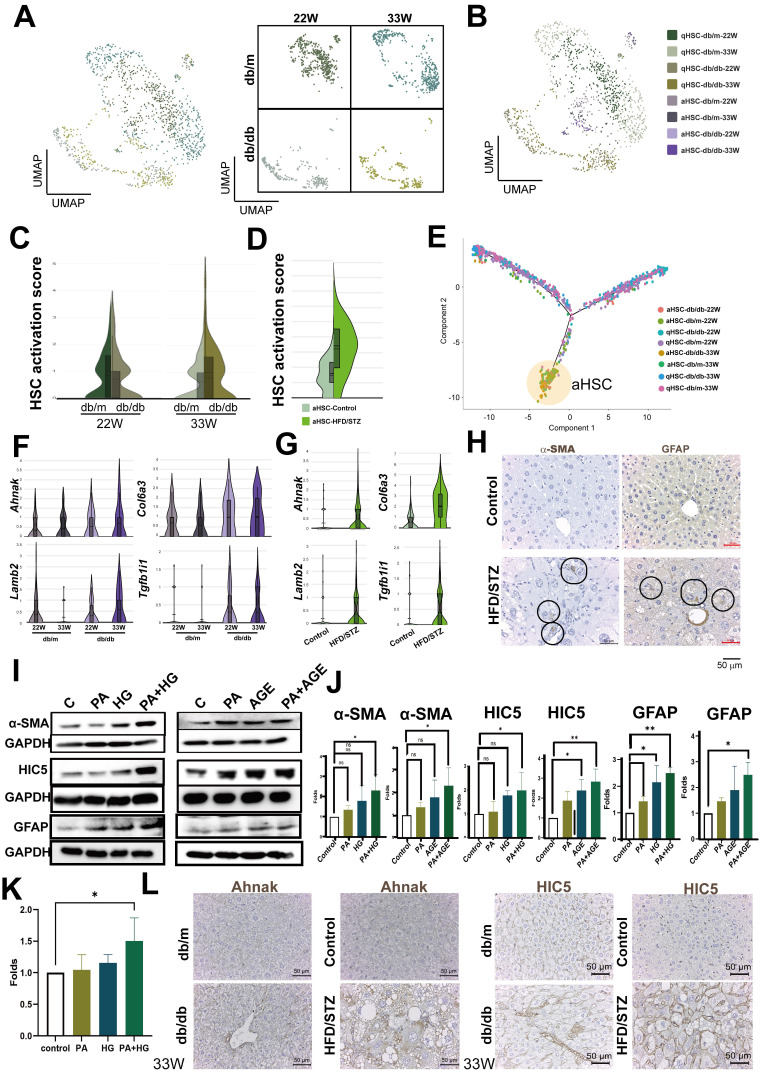
** Increased activation of HSCs at the late-stage of T2DM**. (A) UMAP plot of HSCs in db/m and db/db mice. (B) Distinction between activated and quiescent HSCs (aHSC and qHSC). (C) Higher activation scores in the HSCs of db/db mice at 33 W of age. (D) Increased activation scores in the HSCs of mice with T2DM using the HFD/STZ model. (E) Transition of HSC activation in the liver of db/db mice. Genes associated with the activation of HSCs in db/db mice (F) and HFD/STZ mice (G). (H) Immunohistochemistry staining shows the increment of HSC activation in the liver of mice with T2DM. Circles indicate activated HSCs in the liver sections. (I) PA (palmitic acid) plus high glucose (HG) or AGEs (Advanced Glycation End-products) increased LX-2 activation and expression of α-SMA, HIC5, and GFAP. LX-2 cells were treated with PA (50 μM) under normal glucose conditions (5.5 mM, control) or high glucose conditions (25 mM, HG) for 48 hours. Alternatively, LX-2 cells were treated with PA, AGE (300 μg/ml), or PA plus AGE under a high glucose condition for 48 hours. Protein expression was analyzed using Western blotting. (J) Quantitative results from Western blot analysis. (K) Upregulated Laminin β2 protein in LX2 induced by PA/HG. (L) The IHC of Ahank and HIC5 expression in liver sections. *, p < 0.05; **, p < 0.01. Expression values are presented as mean ± SD.* In vitro* experiments were performed with at least three independent replicates.

Since HSCs play a crucial role in fatty liver, we initially focused on the analysis of HSCs. We annotated the phenotype of HSC as activated HSC (aHSC) or quiescent HSC (qHSC) based on their expression of activation-associated genes (*Tagln*, *Acta2*, *Col1a1,* and *Col1a2*) or quiescence-associated genes (*Lrat*, *Rgs5*, *Ecm1*, *Angpt6*, *Vipr1*, *Guy1a1*, and *Guy1b1*) (Figure 3A, B, Figure S1A, S1B). Compared with db/m mice, the HSC activation score was higher in the HSCs of late-stage diabetic mice, but only a slight increase at the mid-stage (Figure 3C). Consistent with the result of late stage, HSC activation score in the livers of HFD/STZ mice also showed a meaningful increase, compared with the HSC of control mice (Figure 3D). We visualized the transcriptional profiles of the HSCs phenotypic transition using trajectories, and the data indicated a transdifferentiation from qHSC to aHSCs (Figure 3E). As shown in Figure 3F, *Ahnak*, *Col6a3*, *Lamb2*, and *Tgfb1i1*, identified through trajectory analysis as being involved in transdifferentiation, were upregulated in the HSCs of db/db mice at late-stage, compared with the HSCs of db/m mice at 33 weeks of age (Figure 3F, Figure S1C). The upregulation of these genes was also observed in HFD/STZ mice, compared with the HSCs of control mice (Figure 3G,). The IHC of α-SMA and GFAP, two markers for HSC activation, also showed an increase of HSCs in the liver of mice with T2DM in HFD/STZ model (Figure 3H). PA plus HG induced HSC activation (α-SMA and GFAP upregulation) and increased the expression of HIC5 (encoded by *Tgfb1i1*) in LX-2, human HSCs. In addition, PA plus AGE also increased HSC activation and HIC5 protein expression in LX-2 (Figure 3I,J). PA plus HG also increased protein expression of laminin β2 in LX-2 (Figure 3K). The elevated expression of Ahank and HIC5 was also observed in the livers of db/db mice and HFD/STZ mice (Figure 3L). These data showed diabetes-induced activation of HSCs and the expression of related genes were caused by multifactorial stimulation.

### ROS/Txnip system contributed to pathogenic change of hepatocytes in the livers of diabetic mice

**Figure 4:**
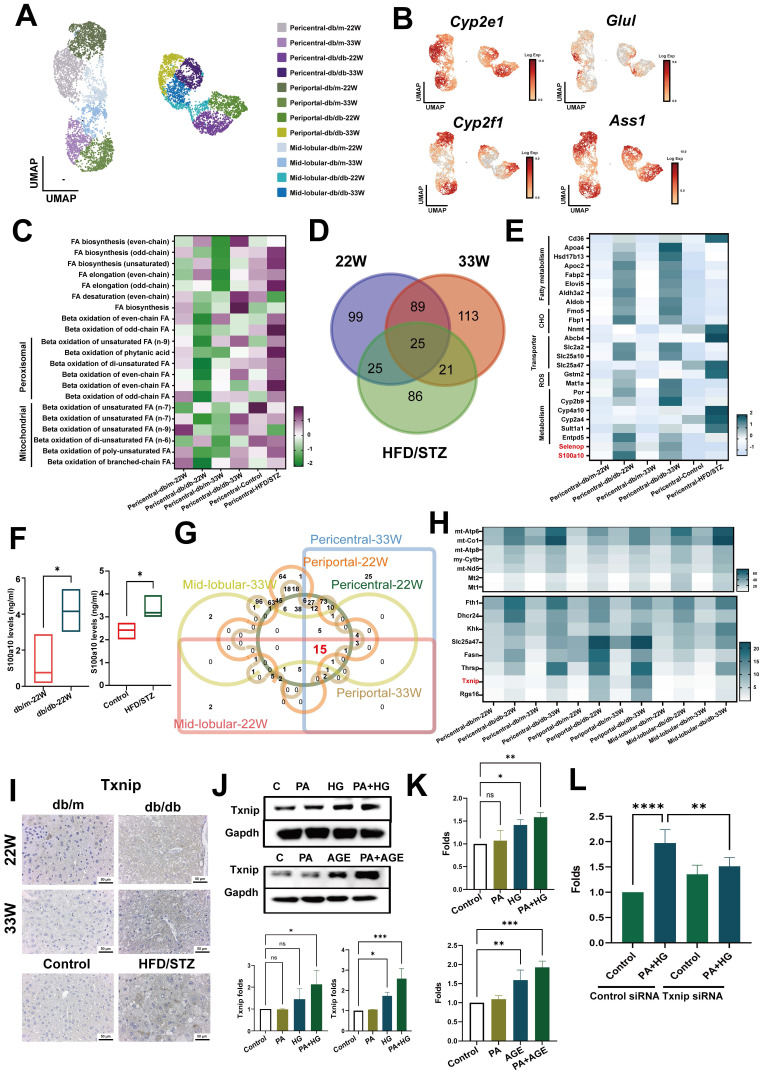
** Txnip contributes to the oxidative stress in hepatocyte of mice with T2DM.** (A) UMAP plot of hepatocyte in db/m and db/db mice. (B) The expression of annotation markers. (C) The changes of pericentral hepatocyte in lipid metabolism at different zones. Venn diagram (D) and heatmap (E) represented the genes regulating the different metabolic profiles at different stages of T2DM. (F) The level of S100A10 protein in the serum of mice. Venn diagram (G) and heatmap (H) indicate pan-genes changed in all hepatocytes of mice with T2DM. (I) IHC staining of Txnip in the liver sections. (K) PA plus HG increased the expression of Txnip in AML-12 hepatocytes. (K) PA plus HG (upper) and PA plus AGE (bottom) increased the production of ROS at 24 hours in AML-12 hepatocytes. (L) Knockdown of *Tnxip* by siRNA transfection decreased ROS production of AML-12 hepatocyte after PA/HG treatment. *, p<0.05; **, p<0.01. Expression values are presented as mean ± SD. *In vitro* experiments were performed with at least three independent replicates.

Next, we evaluated the impact of diabetes on hepatocytes. Liver zonation markers were used to reconstruct the liver zonation, identifying pericentral cells (*Cyp2e1* and *Glul*), periportal cells (*Cyp2f2* and *Ass1*) 16, and the mid-lobular area between the central and portal zones (Figure 4A,B and Figure S2A, S2B). Previous research confirmed that the onset of MAFLD begins in pericentral cells, hence we analyzed changes in the metabolic pathways of pericentral cells using MetaFlux 17, 18. The results showed the primary changes in metabolic pathways were in fatty acid metabolism in pericentral hepatocytes Figure S2C). The elongation of odd chain unsaturated fatty acids significantly increased in both the mid-stage and late-stage of diabetes, and in HFD/STZ mice. Correspondingly, the β-oxidation of odd chain fatty acids, and both peroxisomal and mitochondrial fatty acid metabolism were meaningfully elevated in the more severe late stages of T2DM or in HFD/STZ model (Figure 4C). Intersection of DEG of pericentral hepatocyte of mice with mid-stage, late-stage and HFD/STZ gene profiles revealed 25 genes (Figure 4D), including fatty acid and carbohydrate metabolism, transporter, xenobiotic metabolism, and two soluble factors (*S100a10* and *Selenop*) were upregulated in the pericentral hepatocytes of mice with T2DM (Figure 4E). ELISA data indicated that elevated levels of the S100A10 protein were present in the sera of mice with T2DM across two different murine models (Figure 4F).

To explore the key factors contributing to liver damage caused by diabetes, we assessed its impact on entire liver area by intersecting DEGs of hepatocytes across all liver zones. The results revealed that 15 genes were upregulated in all zones of the liver in db/db mice (Figure 4G,H). Among these,* Txnip* was also upregulated in all zones of the liver in the HFD/STZ model (Figure S2E). IHC further supported that Txnip was significantly overexpressed in the livers of both types of diabetic mice (Figure 4I). *In vitro* data showed that PA plus HG or PA plus AGE enhanced the levels of Txnip in AML-12 liver cells (Figure 4J). Moreover, both PA plus HG and PA plus AGE also increased the level of ROS in AML-12 cells after 24 hours, with an even greater increase at 48 h (Figure 4K and Figure S2F). Knockdown of *Txnip* decreased ROS production in AML-12 cells induced by PA plus HG (Figure 4K and Figure S2G). These data demonstrated that the ROS/Txnip axis was involved in HG-mediated pathogenic changes in fatty liver.

### Increased capillarization of endothelial cells in the liver of diabetic mice

**Figure 5:**
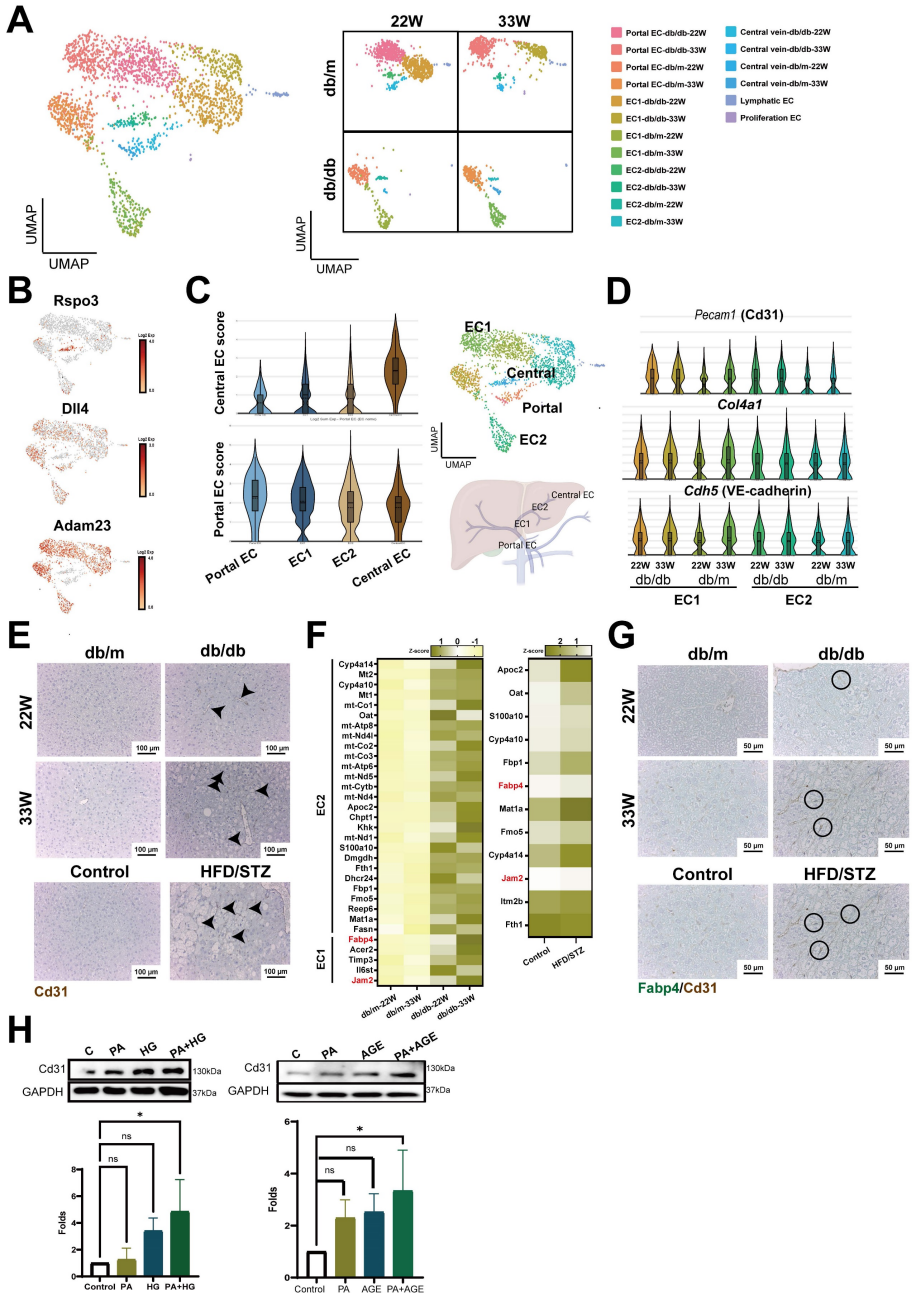
** Enhanced capillarization of endothelial cells (ECs) in the livers of diabetic mice.** (A) UMAP plot of ECs in db/m and db/db mice. (B) The distribution of specific genes associated with central and portal EC. (C) Central or Portal EC score in all cell clusters of ECs. (D) The expression of capillarization markers in the cell clusters of liver EC. (E) IHC represents CD31^+^ EC in the liver of mice with T2DM. (F) Heatmap of upregulated genes in EC1 and EC2 clusters in mice at 22W and 33W of age. (G) IHC represents CD31^+^FABP4^+^ ECs in the liver of mice with T2DM. (H) PA (palmitic acid) plus high glucose (HG) increased the level of CD31 in HHSECs. HHSECs were treated with PA (50 μM) under normal glucose conditions (5.5 mM) or high glucose conditions (25 mM) for 48 hours. Protein expression was analyzed using Western blotting. (I) Quantitative results from Western blot analysis. *, p < 0.05; Expression values are presented as mean ± SD. *In vitro* experiments were performed with at least three independent replicates.

Liver ECs, including liver sinusoidal endothelial cells (LSECs), vascular endothelial cells, and lymphatic endothelial cells, are crucial for maintaining liver homeostasis (5. Therefore, we analyzed the impact of diabetes on liver ECs. In addition to identifying lymphatic and proliferative endothelial cells, we categorized endothelial cells into portal ECs *(Adam23*+), central ECs (*Rspo3+*), and distinct clusters, EC1 and EC2, utilizing liver annotation markers (Figure 5A,B).

Gene expression analysis further delineated that the EC1 cluster closely aligned with the portal ECs, whereas the EC2 cluster showed greater similarity to the central ECs (Figure 5C). By analysis of differential expression genes between db/m and db/db mice liver ECs, it was observed that several genes critical for vascular structure, including* Pecam1* (encoded Cd31), *Cdh5* (encoded VE-cadherin), and *Col4a1*, exhibited heightened expression in both EC1 and EC2 clusters of db/db mice (Figure 5D). IHC results showed increased expression of CD31 in the liver of mice with T2DM at both mid and late stages. This increase was also observed in the liver of HFD/STZ diabetic mice (Figure 5E). In addition, tight junction *Jam*2 and fatty acid binding protein, *Fabp4*, were also notably upregulated in ECs of mice with T2DM across transgenic mice and HFD/STZ models (Figure 5F). All genes associated with capillarization were consistently upregulated in the liver ECs of diabetic mice in HFD/STZ model Figure S3A,B). Double-stained IHC results also showed a high expression of FABP4 in the *Cd31^+^* ECs of diabetic mouse livers (Figure 5G). *In vitro* results indicated that both PA plus HG and PA plus AGE increased the expression of CD31 in HHSECs, demonstrating that the presence of high glucose and fatty acids enhances the capillarization of liver (Figure 5H).

### *Cd36^+^* Kupffer cells (KCs) possessed inflammatory phenotype in the livers of diabetic mice

**Figure 6:**
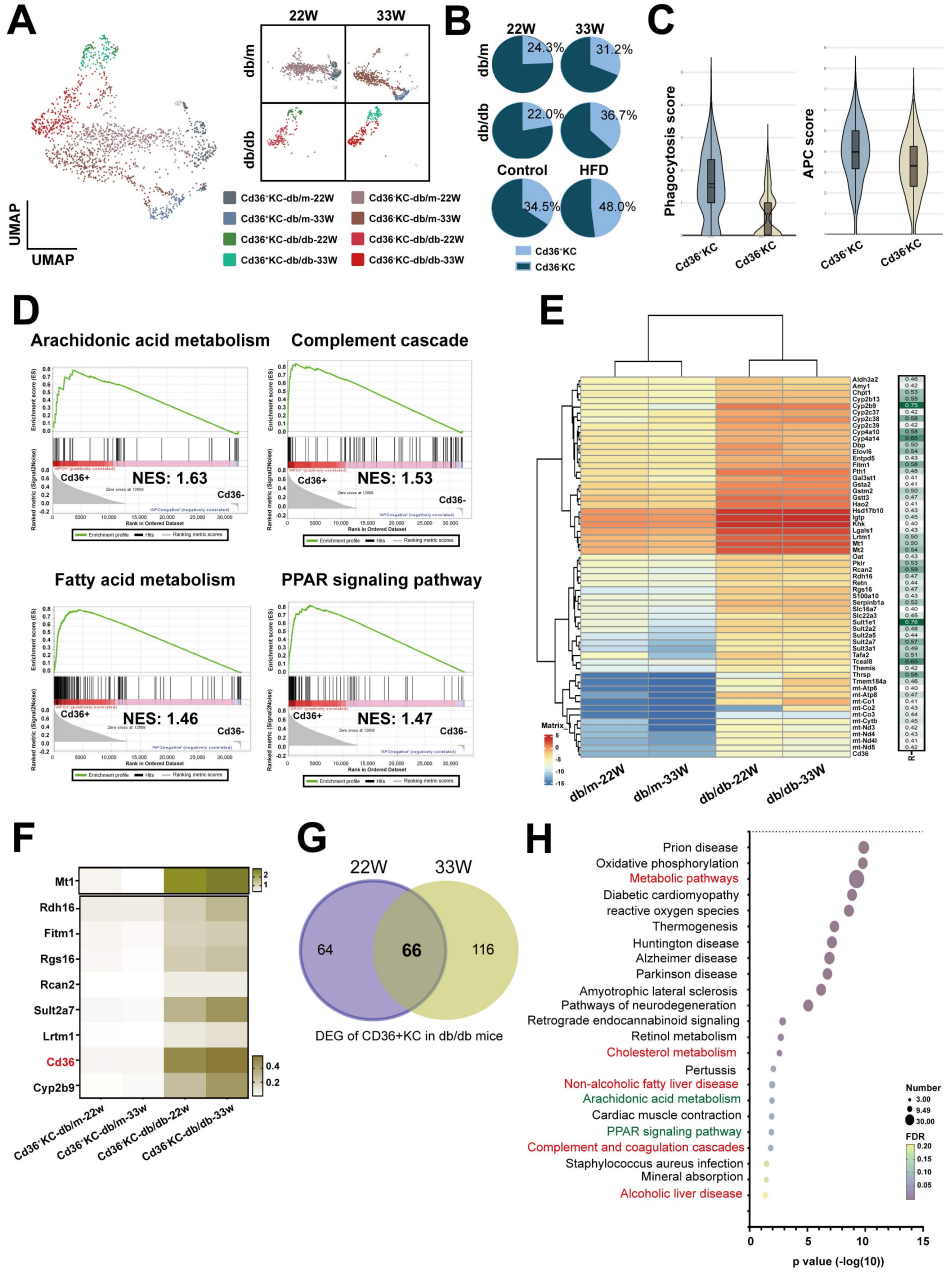
** Increased *Cd36*^+^ KCs infiltration in the liver of mice with T2DM.** (A) UMAP plot of KCs in db/m and db/db mice. (B) The ratio of *Cd36*^-^ and *Cd36*^+^ KCs in the livers of db/m and db/db mice at 22W and 33W of age. (C) The APC and phagocytosis score of *Cd36*^+^ and *Cd36*^-^ KCs. (D) GSEA of *Cd36*^+^ and *Cd36*^-^ KCs. (E) The genes positively correlated with *Cd36* expression in KC cells. (F) Heatmap of genes upregulated in the KCs of mice with T2DM. (G) Venn diagram of DEG of *Cd36^+^* KCs of db/db mice with mid- or late-stages T2DM. (H) The KEGG pathways of 66 genes upregulated in *Cd36*^+^ KCs of mice with T2DM. Abbreviations: APC, antigen-presenting cell; GSEA, gene set enrichment analysis; DEG, differentially expressed gene.

KCs are specialized macrophages located in the liver and play a significant role in liver homeostasis and immune regulation. We analyzed KCs, and the results identified two subsets based on the level of *Cd36* expression (Figure 6A). Notably, the cell number of *Cd36^+^* KCs was found to be increased in diabetic mice across both models (Figure 6B). Functional analysis showed that higher scores of phagocytosis and antigen-presenting function were found in* Cd36^+^* KCs, compared with *Cd36^-^* KCs (Figure 6C). GSEA analysis revealed that *Cd36^+^* KCs were associated with inflammatory pathways (arachidonic acid metabolism and complement cascade) and lipid-related pathways (fatty acid metabolism and PPAR signaling pathway) (Figure 6D). Correlation analysis of *Cd36* expression shown 58 genes had a high positive correlation in *CD36^+^* KCs (r > 0.4, p < 0.0001) (Figure 6E). Eight genes (*Mt1*, *Rdh16*, *Fitm1*, *Rgs16*, *Rcan2, Sult2a7, Lrtm1*, and *Cyp2b9*) had expression patterns similar to *Cd36*, which exhibited significantly higher expression levels within *Cd36^+^* KCs in db/db mice compared with db/m mice (Figure 6F).

Next, we explored the differences in* Cd36*^+^ KCs under the influence of diabetes and non-diabetes conditions. We compared the DEGs of *Cd36^+^* KCs between db/m mice and db/db mice, which identifying 66 genes with significantly increased expression at both mid- and late-stages (Figure 6G). KEGG analysis indicated that associations with metabolism, arachidonic acid metabolism, PPAR, and complement pathway, consistent with GSEA results (Figure 6H). These findings suggested that diabetes modified KCs metabolism, enhancing their transition to a more active state.

### The cell-cell-communications in the liver of diabetic mice

**Figure 7:**
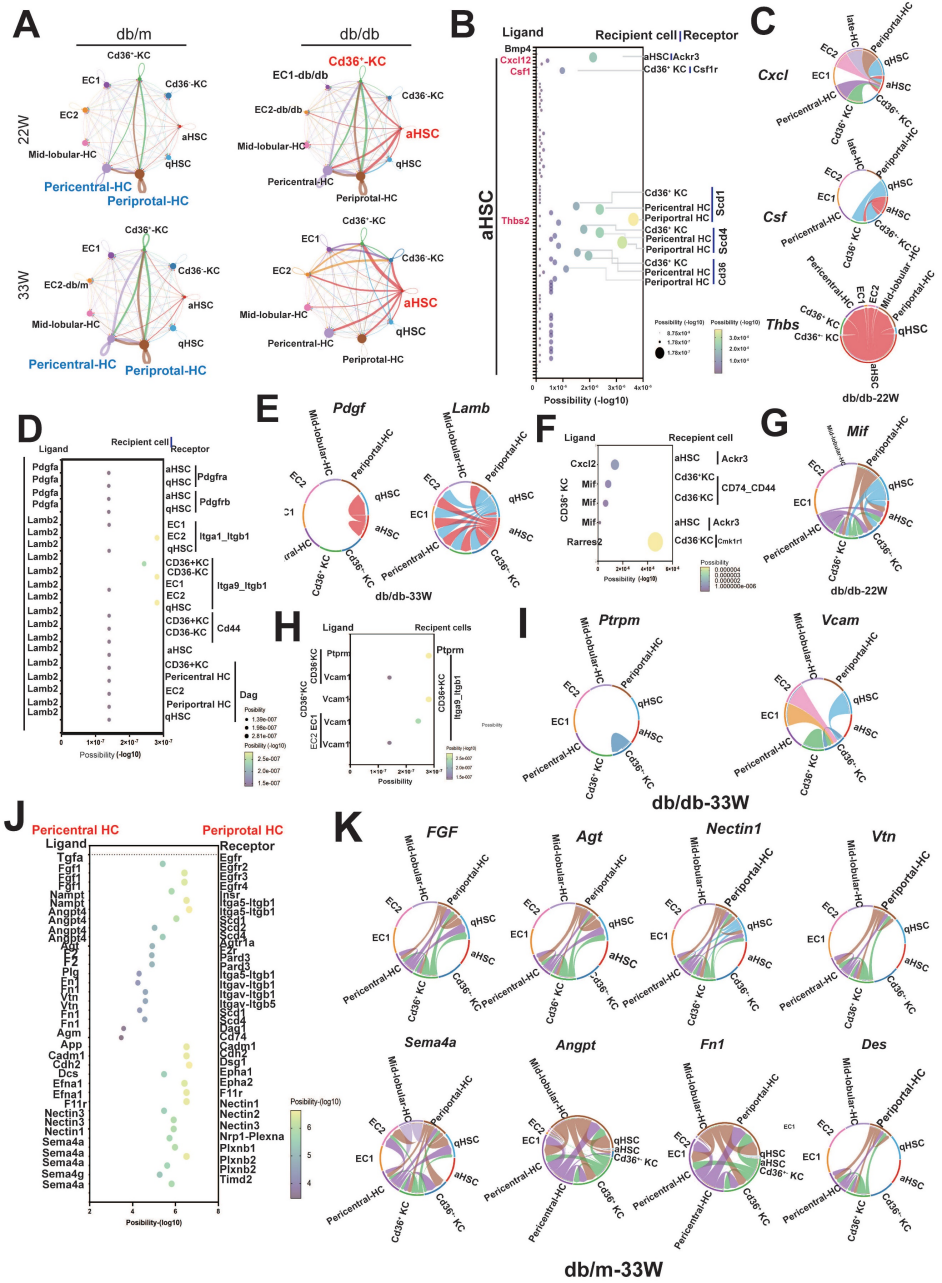
** The cell-cell interaction in the hepatic microenvironment of mice with T2DM.** (A) The cell-cell communications in the livers of db/m and db/db mice at 22W and 33W of age. (B) The interaction of aHSCs with hepatocytes and KCs in the livers of mice at 22W of age. (C) The chord diagram visualizes the cell-cell communication in *Cxcl*,* Csf,* and* Thbs2*. (D) The cell communication of aHSCs with other cell types in the livers of mice at 33W of age. (E) The chord diagram visualizes the cell-cell communication in *Pdgf* and *Lamb*. (F) The intercellular signaling between *Cd36*^+^ KCs with other cell types in livers of mice at 22W of age. (G) The chord diagram represents the interaction mediated by *Mif*. (H) The intercellular signaling between *Cd36*^+^ KCs with other cell types in the liver of mice at 33W of age. (I) The chord diagram represents the interaction mediated by *Ptprm* and *Vcam*. (J) Loss of intercellular signaling in the hepatocyte in the mice with T2DM. (K) The chord diagram represents the signaling pathways loss in the liver of mice with T2DM.

Cell-cell interactions play a critical role in the initiation and progression of various diseases, including diabetes. Therefore, we investigated the signaling networks in the liver underlying diabetic pathogenic condition, instead of physical status. In mid-stage diabetes, an increase in cell-cell interactions was found in the livers of diabetic mice primarily originating from aHSCs and* Cd36^+^* KCs, as revealed by ligand-receptor pair analysis. Conversely, in late-stage diabetes, the predominant signaling was observed to originate from aHSCs (Figure 7A). As shown in Figure 7B and 7C, autocrine interaction within aHSCs was mediated by the *Cxcl12-Ackr3* axis. aHSCs also engaged in the interactions with *Cd36^+^* KCs and hepatocytes through the *Thbs2-Scd1/Scd4* or *Thbs2-Cd36* axis. In contrast to mid-stage diabetes, aHSCs in late-stage diabetes regulated their phenotype via the *Pdgfa-Pdgfra/b* axis (Figure 7D). Furthermore, through *Lamb2* secretion, aHSCs also regulated ECs, hepatocytes, and *Cd36^+^* KCs (Figure 7E). In addition to aHSCs in the livers of diabetic mice, *Cd36^+^* KCs were also found to influence themselves or aHSCs via the *Cxcl12-Ackr3* and *Mif-Cd74-Cd44* pathways (Figure 7F,G). Furthermore, in the late-stage, ECs and* Cd36^-^* KCs were also able to engage in cell-cell communication via *Vcam1* and *Cd36^+^* KCs (Figure 7H,7I).

Alongside the increased cell-cell interactions observed in diabetic liver, some interactions were notably diminished, especially those involving communications within hepatocytes. As shown in Figure 7J, the interactions between periportal and pericentral hepatocytes, mediated by* Fgf*, *Agt, Nectin1, Vtn*, *Sema4a*, *Angpt*, *Fn1*, and *Des*, were lost in the livers of mice with diabetes at the late-stage (Figure 7J,K).

## Discussion

Epidemiological studies reveal that the prevalence of MAFLD, including severe stages like advanced fibrosis, is notably higher in patients with T2DM than in the general population (19, 20. T2DM-related MAFLD not only escalates the risk of severe liver complications and increased mortality but also correlates with various comorbidities such as diabetic cardiovascular disease and nephropathy 21, 22. However, significant gaps remain in understanding the complex pathogenic mechanisms of MAFLD in T2DM. Due to the heterogeneity of the liver and phenotypic transitions among specific cell types, advanced high-resolution methodologies such as scRNA-seq is crucial for elucidating the pathogenic mechanisms of organ damage driven by hyperglycemia. Through the application of scRNA-seq, we have delineated the cellular heterogeneity within the liver, documenting variations in hepatocytes, HSCs, LSECs and KCs. Moreover, our research has unveiled a previously unidentified subset of liver resident macrophages, *Cd36^+^* KCs, characterized by an inflammatory phenotype. This discovery highlights the need for further research to understand its role in the progression of MAFLD underlying T2DM.

Hepatic steatosis occurs due to the imbalance in lipid metabolism within hepatocytes, the major type of cells in the liver 23. This condition arises predominantly from excessive fatty acid (FA) uptake from the bloodstream and increased *de novo* lipogenesis 24. Elevated levels of FAs activate inflammatory pathways, potentially leading to liver damage. Furthermore, disrupted mitochondrial β-oxidation leads to alternative oxidation in peroxisomes and mitochondria, producing high levels of ROS and other harmful by-products. Despite previous studies indicating enhanced FA oxidation capabilities in specific animal models 25, 26, some study reveals that complete FA oxidation in hepatic mitochondria is significantly reduced by approximately 40-50% in individuals with NASH compared with controls with normal liver histology 27. This reduction correlates with increased ROS production and decreased mitochondrial biogenesis and mitophagy markers, highlighting the complex and controversial nature of FA oxidation dysregulation in hepatic steatosis 28. Our results indicate that in the mid-stage of the disease, both mitochondrial and peroxisomal β-oxidation are significantly reduced in db/db mice compared with db/m ones. However, in the more severe late-stage or in the HFD-STZ model, both mitochondrial and peroxisomal β-oxidation are markedly increased, accompanied by higher levels of *de novo* lipogenesis, including in FA biosynthesis, elongation, and desaturation. In addition, an increase in ROS was also observed in *in vitro* experimental models, such as AML-12 hepatocyte treated with PA/HG or PA/AGE. Moreover, TXNIP*,* an inhibitory protein that binds to endogenous antioxidant thioredoxin, was found to be upregulated across all zonation areas of hepatocytes, and knockdown of Txnip prevented PA plus HG-mediated ROS production. This suggests that elevated TXNIP levels inhibit the antioxidative function of thioredoxin, leading to an accumulation of ROS and increased cellular stress.

HSC activation and fibrosis are considered key factors that lead to MAFLD progression 29. Activated HSCs (aHSCs) are the primary producers of extracellular matrix (ECM) proteins in the liver, generating substantial quantities of collagen and other ECM components that disrupt normal liver functions 30. Growing evidences demonstrated that targeting aHSCs could prevent or reverse liver fibrosis and these strategies include developing medications that either inhibit the activation of HSCs or encourage their apoptosis 31. Our results showed that HSC activation occurs in the late-stage or in severe HFD/STZ diabetic model. In addition to the increased expression of the ECM protein COL4A6, LAMB2, and HIC5 (encoded by *Tgfb1i1*) are also present in the livers of mice with late-stage diabetes and HFD/STZ. PA, a major C16 saturated FA, plus HG increased HSC activation and laminin β2 expression, indicating that multiple stimuli are required to activate HSCs. In addition, ligand-receptor pair analysis revealed that aHSC could interact with hepatocytes and *Cd36^+^* KCs via the *Lamb2/Dag* axis, which have been demonstrated to be associated with liver fibrosis 32. HIC5 has also been reported to contribute to liver fibrosis induced by Ccl4 by suppressing the expression of Smad7 and enhancing the pro-fibrotic activity of TGF-β 33. Moreover, the* Pdgf/Pdgfr* axis, a well-known pro-fibrosis pathway, was found to dominate the interactions between aHSC-qHSC and aHSC-aHSC, indicating the existence of a positive feedback loop through an autocrine model in diabetes-associated MAFLD. Taken together, currently our results have identified several new pathological molecules secreted by aHSCs that are involved in diabetes-related MAFLD and even NASH, including *Lamb2* and *Tgfb1i1*. These findings merit further research and exploration as potential targets for preventing severe liver fibrosis.

LSECs are specialized by their unique feature of having organized fenestrae and lacking a basement membrane 34. In some pathological conditions, these cells undergo a transformation known as "capillarization". During this process, LSECs lose their fenestrae and develop a basement membrane which unusually precedes the onset of fibrosis. The inducers and pathogenic factors for liver vessel capillarization are not fully identified. Excessive dietary macronutrients, including lipids and carbohydrates, may contribute to the abnormal alterations in LSECs 35. High glucose levels or oxidized low-density lipoprotein exposure leads to the contraction and subsequent defenestration of fenestrae in rat LSECs by integrin/FAK signaling cascade 36, 37. Previously studies have indicated that FABP4 is involved in the capillarization of LSECs, as demonstrated using a *Fabp4* knockout mouse model 38. Our findings indicate that capillarization of ECs was observed in the liver of diabetic mice at the mid-stage, as evidenced by elevated levels of *Cd31* and the basement membrane matrix component *Col4a1*. Interestingly, the tight junction* Jam2* was also upregulated in the ECs of mice with T2DM. Capillarization markers *Cd31* and *Fabp4*, were highly co-expressed in the ECs of diabetic mice in both transgenic and HFD/STZ models, suggesting a potential role for* Fabp4* in contributing to liver capillarization under diabetic conditions. Additionally, the combination of fatty acid, PA, with HG or AGEs heightened the expression of CD31 in human hepatic sinusoidal endothelial cells *in vitro*. This supports the hypothesis that an excess of fats, sugars, and their metabolites may be key factors driving liver pathology. FABP4, as implicated in diabetes-induced MAFLD, represents a promising target for future studies aimed at developing therapeutic interventions for MAFLD or strategies to prevent the progression to NASH and liver fibrosis.

**Figure 8:**
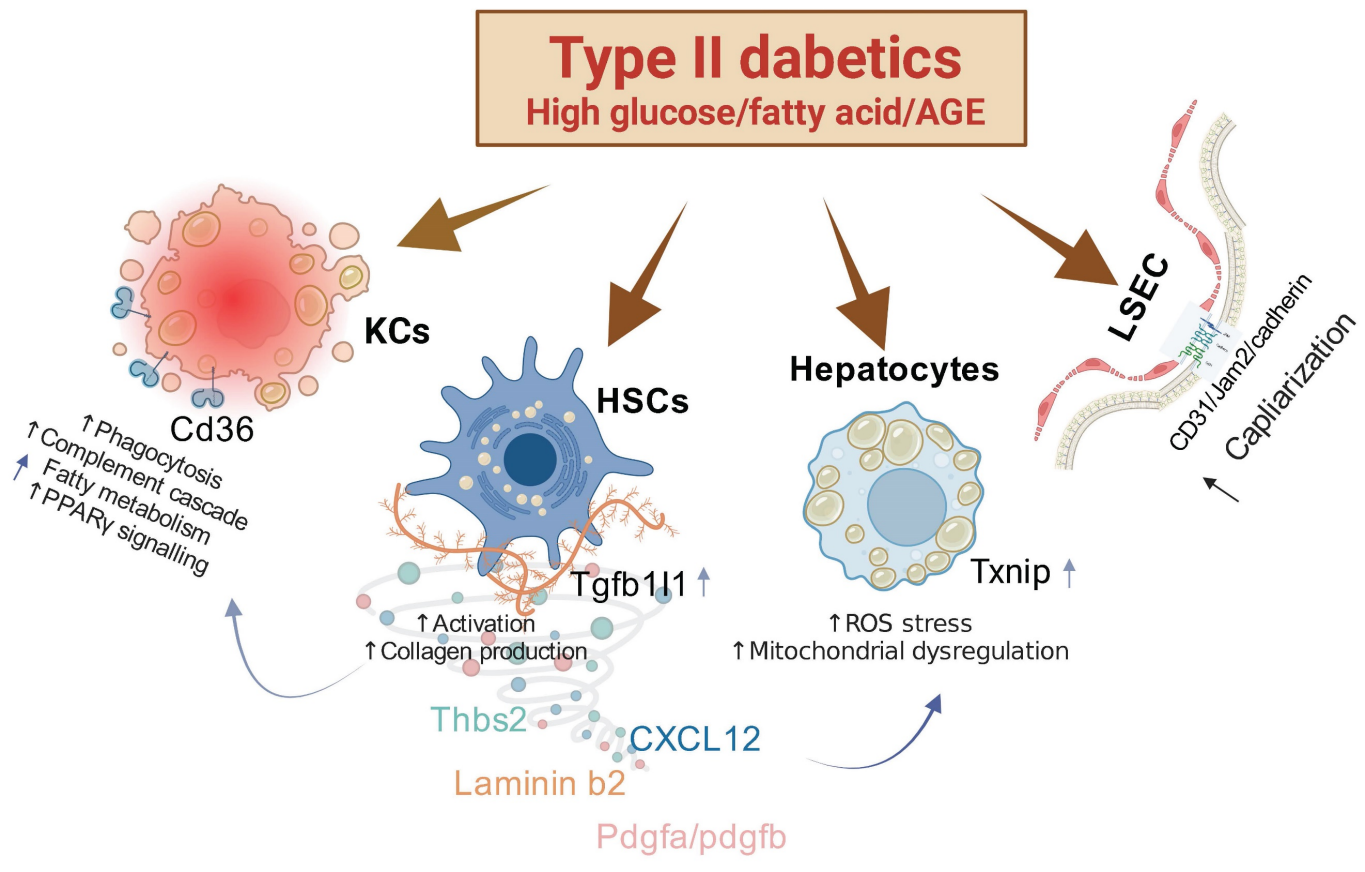
** Mechanisms of Action in Liver Pathophysiology Associated with Type II Diabetes.** This figure illustrates several key pathological changes linked to type II diabetes in the liver. First, the overactivity of hepatic stellate cells (HSCs) is notably enhanced by the protein HIC5. Second, the overexpression of TXNIP compromises the liver's antioxidant defenses, leading to an increase in reactive oxygen species (ROS) stress and subsequent alterations in hepatocyte function. Third, an increase in the capillarization of liver sinusoidal endothelial cells (LSECs) is associated with heightened expression of FABP4. These pathological shifts are particularly severe under combined conditions of high fat and high glucose or high fat and advanced glycation end-products (AGEs), indicating a synergistic exacerbation of liver pathology under these dietary stresses.

In our comprehensive investigation of the pathophysiological changes in the liver induced by diabetes, we have identified several key findings. First, we discovered that the pathological molecule *Tgfb1i1* (HIC5) enhances the excessive activation of HSCs. Second, overexpression of TXNIP in the liver inhibits anti-oxidation mechanisms, leading to increased ROS stress and subsequent alterations in hepatocytes. Third, the capillarization of LSECs is associated with the overexpression of FABP4. These pathophysiological changes are stimulated under conditions of both high fat and high glucose or high fat and AGEs, indicating that these conditions synergistically exacerbate liver pathology (Figure 8). Therefore, simultaneously controlling lipids and blood glucose or targeting these specific molecules may represent a novel and effective strategy for preventing diabetic liver disease.

## Supplementary Material

Supplementary figures and table.
