# Supplementary material for: Reconstruction of the Hepatic Microenvironment and Pathological Changes Underlying Type II Diabetes through Single-Cell RNA Sequencing
Source: Int J Biol Sci. 2024 Oct 14;20(14):5531–47. doi: 10.7150/ijbs.99176 (PMC11528452; doi:10.7150/ijbs.99176)
Supplement: Supplementary file 1 — Supplementary figures and table. [file ijbsv20p5531s1.pdf]

sTable 1. The antibodies used in this study

**Western blot**		
Protein	Catalog number	Diluted ratio
**abcam**		
TXNIP	ab188865	1:1000
CD36	ab252923	1:1000
**Proteintech**		
HIC5	10565-1-AP	1:3000
**Cell signaling**		
CD31	3528S	1:1000
**Sigma**		
α-SMA	A5228	1:1000
**EMD Millipore**		
GAPDH	MAB374	1:3000

**IHC**		
Protein	Catalog number	Diluted ratio
**Abcam**		
TXNIP	ab188865	50X 2hr
CD31	ab28364	50X
FABP4	ab92501	100X
α-SMA	ab7817	1000X
GFAP	Ab7260	100X
**Proteintech**		
HIC5	10565-1-AP	50X
AHNAK	16637-1-AP	200X

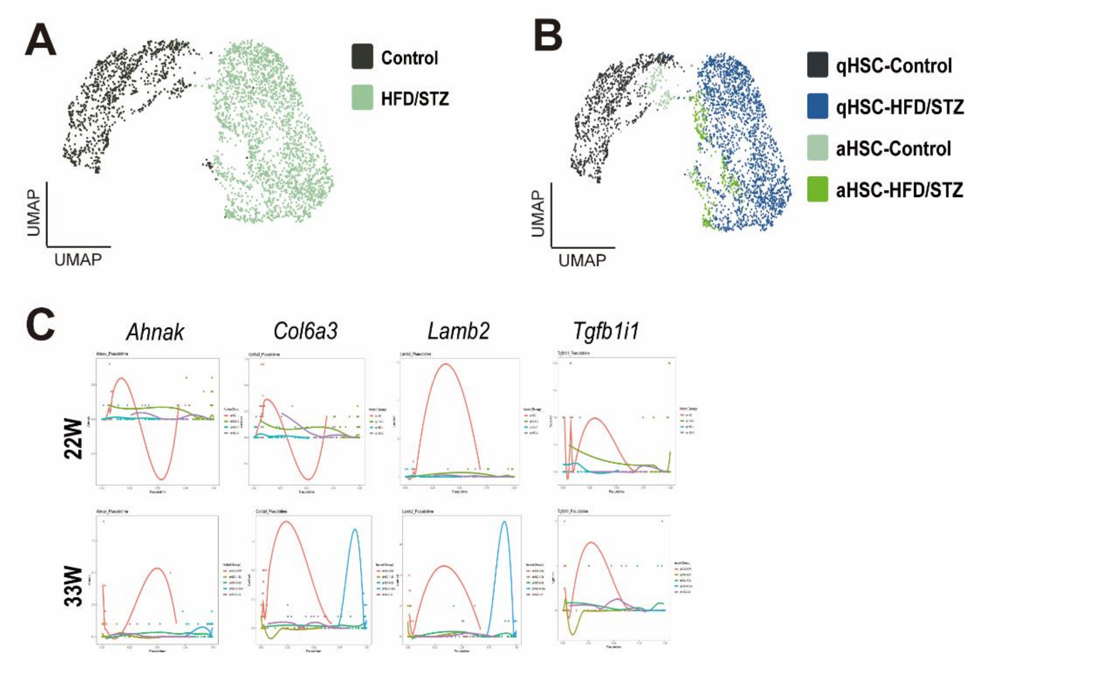

Figure S1. The genes contributed to the HSC activation. (A) UMAP plot of HSCs in control and HFD/STZ mice. (B) The distinction between activated and quiescent HSCs (aHSCs and qHSCs). (C) The gene associated with the activation of HSCs.

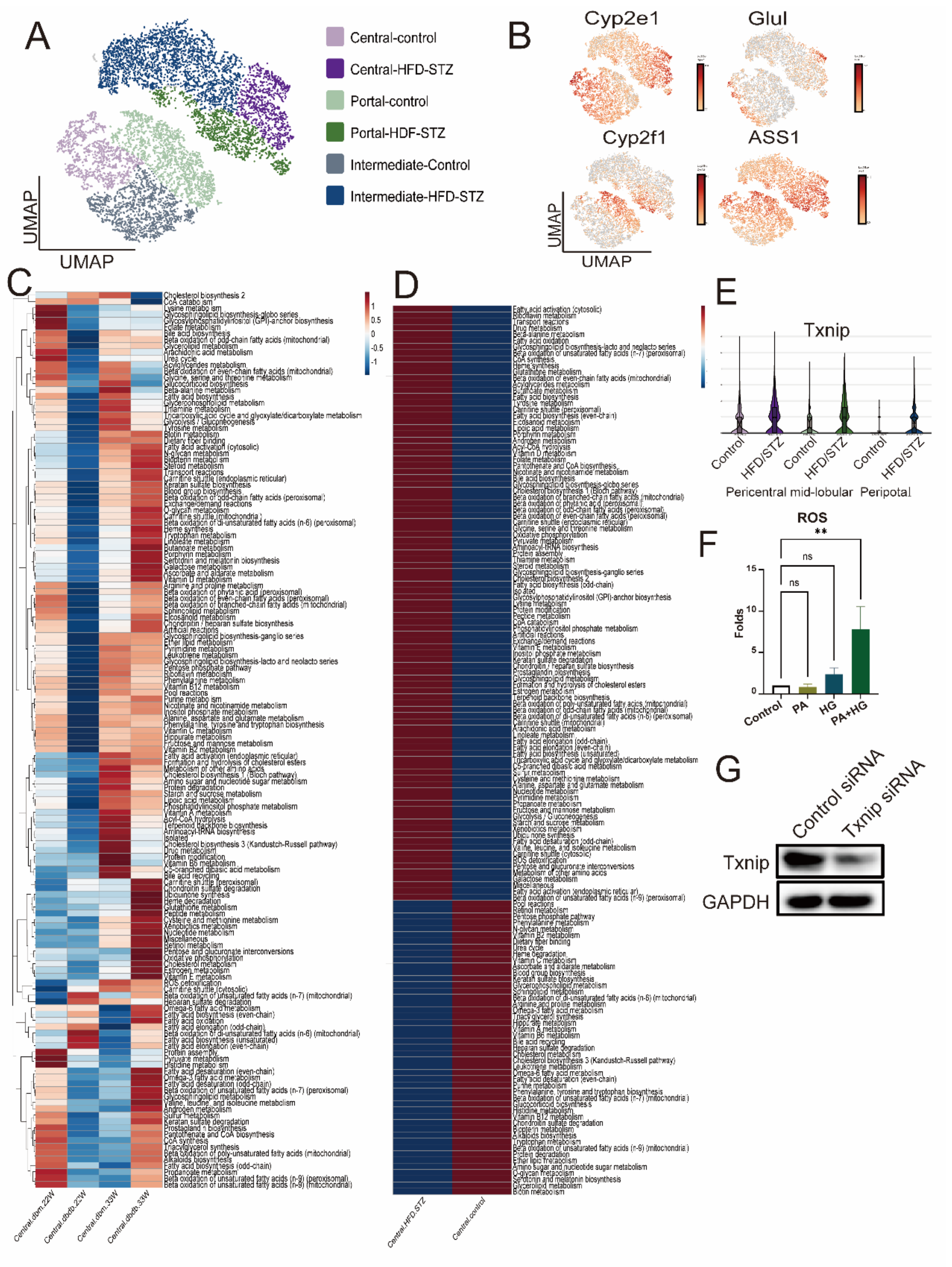

Figure S2. The changes of lipid metabolism of hepatocytes in the HFD/STZ model. (A) UMAP plot of hepatocytes in db/m and db/db mice. (B) The expression of annotation markers. Heatmap represented the change of various metabolism pathways in hepatocytes of db/db mice (C) and mice treated with HFD/STZ (D). (E) The expression of Txnip in hepatocytes of mice treated with HFD/STZ. (F) The effect of PA or PA plus HG in the ROS production after 48 h incubation. (G) Knockdwon efficacy Tnxip siRNA in AML-12 cells.

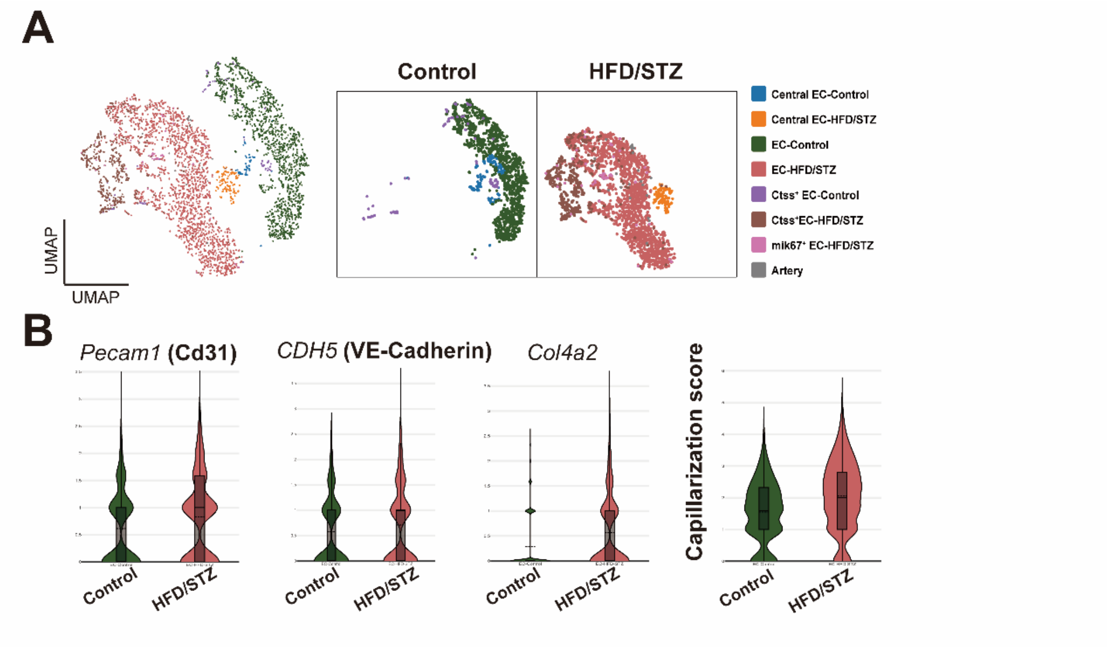

Figure S3. Enhanced capillarization of endothelial cells (ECs) in the livers of diabetic mice using HFD/STZ model. (A) UMAP plot of ECs in control and HFD/STZ mice. (B) The expression of capillarization markers in the cell clusters of liver ECs.
